# The Ratio of Preoperative Serum Biomarkers Predicts Prognosis in Patients With Oral Squamous Cell Carcinoma

**DOI:** 10.3389/fonc.2021.719513

**Published:** 2021-09-06

**Authors:** Meng Ding, Yuxian Song, Junyan Jing, Mei Tian, Liang Ding, Qiang Li, Chongchong Zhou, Heng Dong, Yanhong Ni, Yongbin Mou

**Affiliations:** ^1^Nanjing Stomatological Hospital, Medical School of Nanjing University, Nanjing, China; ^2^Central Laboratory, Nanjing Stomatological Hospital, Medical School of Nanjing University, Nanjing, China; ^3^Department of Oral Implantology, Nanjing Stomatological Hospital, Medical School of Nanjing University, Nanjing, China

**Keywords:** oral squamous cell carcinoma, neutrophil-to-lymphocyte ratio, lymphocyte-to-monocyte ratio, neutrophil-to-white blood cell ratio, lymphocyte-to-white blood cell ratio, overall survival, disease-free survival, metastasis-free survival

## Abstract

**Background:**

Dynamic changes in circulating immune-inflammatory cells have been regarded as simple and convenient prognostic biomarkers in various cancers. However, studies on the prognostic values of their ratios in oral squamous cell carcinoma (OSCC) remain limited.

**Materials and Methods:**

A total of 493 OSCC patients were included in the present study. Here, we investigated the prognostic values of the neutrophil-to-lymphocyte ratio (NLR), lymphocyte-to-monocyte ratio (LMR), neutrophil-to-white blood cell ratio (NWR), and lymphocyte-to-white blood cell ratio (LWR) in OSCC. The correlations of the NLR, LMR, NWR, and LWR with clinicopathological characteristics were statistically analyzed using the Chi-square test, Kaplan-Meier curves, and univariate and multivariate Cox regression models.

**Result:**

Kaplan-Meier analyses revealed that OSCC patients with a high LMR and low NWR had prolonged overall survival (OS, *P*<0.001) and disease-free survival (DFS, *P*<0.001 and *P*=0.003, respectively), but there were no significant differences in metastasis-free survival (MFS, *P*=0.053 and *P*=0.052, respectively). In contrary, a high NLR and low LWR were associated with poor OS (*P*<0.001 and *P*=0.0016, respectively), DFS (*P*=0.0014 and 0.0012, respectively) and MFS (*P*=0.021 and 0.008, respectively). Additionally, Cox multivariate analyses showed that the LMR was an independent prognostic factor for both OS (*P*=0.007) and DFS (*P=*0.017), while the LWR was an independent prognostic factor for MFS (*P*=0.009).

**Conclusion:**

Preoperative NLR, LMR, NWR, and LWR in the peripheral blood are significant prognostic factors for OSCC and might be helpful in predicting OSCC progression.

## Introduction

Oral squamous cell carcinoma (OSCC) refers to a malignant tumor that originates from the mouth and is dominated by squamous cells (1). Cancer cells can occur in the gingiva, hard palate, tongue, buccal mucosa, lip and other organs, and the number of confirmed OSCC cases is predicted to rise to 856,000 cases by 2035 worldwide (2). Although many therapeutic strategies for OSCC have shown promising effects in the treatment of OSCC in recent decades, the 5-year survival rate remains at approximately 65% (3). In addition, the survival rates of patients are affected by the stage of cancer progression. For localized OSCC, the 5-year survival rate is 84% but decreases to 65% in regional cases and to 39% if distant metastasis is present (4). In other words, a high survival rate relies largely on early detection, which is the key to improving the quality of life of OSCC patients. To avoid distant metastasis of OSCC due to a missed diagnosis, local and precise approaches are urgently needed to prevent, screen, and intervene in OSCC.

The traditional tumor staging system is based on the primary tumor classification (T), quantification of nodal metastasis (N), and presence of distant metastasis (M) (5). However, patients with the same TNM stages often show significantly different prognoses, which indicates that the TNM staging system remains far from optimal in predicting OSCC outcomes, especially the clinical TNM (cTNM) (6, 7), which largely relies on the radiological evaluation. It is well known that the radiological examination can be influenced by the limitations of imaging techniques or technicians, which may result in inaccurate classification (8). Thus, some studies considered introducing a prognostic evaluation using serum biomarkers (such as neutrophils, lymphocytes, and monocytes) to optimally stratify patients, select treatment strategies and predict prognosis in the clinic (9, 10). The main strength of the novel method is that the information can be obtained from routine blood tests before surgery, without any extra effort, making it a simple, economical, and real-time prediction tool.

Numerous studies have indicated that changes in circulating immune-inflammatory cells, such as monocytes, lymphocytes, neutrophils, and platelets, in the peripheral blood could be novel prognostic biomarkers in soft tissue sarcomas or oropharyngeal cancer (10, 11). However, whether assessing the circulating immune-inflammatory in peripheral blood is suitable for OSCC is still unclear. Tsai et al. studied 202 OSCC patients and found that the pretreatment circulating monocyte count increased with advanced clinical stage (12). In a retrospective study of 309 patients with OSCC, a high platelet count, high neutrophil count and low lymphocyte count were found to be associated with reduced overall survival (OS) and disease-free survival (DFS) (13). Some studies have also indicated that an increased pretreatment neutrophil-to-lymphocyte ratio (NLR) is associated with poor outcomes in hepatocellular carcinoma, colorectal cancer, endometrial cancer, and gastric cancer (14–17). However, few studies have reported the prognostic values of the ratios of immune-inflammatory cells, such as the NLR, lymphocyte-to-monocyte ratio (LMR), neutrophil-to-white blood cell ratio (NWR), and lymphocyte-to-white blood cell ratio (LWR), especially in patients with OSCC. Moreover, previous studies have focused on the relationship between serum biomarkers and OS or DFS, but the predictive capacity of these biomarkers for metastasis has not yet been reported.

Therefore, the current study aimed to evaluate the association of the NLR, LMR, NWR, and LWR with clinicopathological characteristics, as well as their prognostic value for OS, DFS, and MFS in OSCC patients.

## Materials and Methods

### Inclusion and Exclusion Criteria of Participants

The clinical data used in the present study were obtained from a database that collected demographic data, clinical characteristics, treatments, and follow-ups of all patients with OSCC who were primary diagnosed and surgically treated at Nanjing Stomatological Hospital from 2012 to 2015.

The inclusion criteria for the study were as follows: (1) primary OSCC without any previous treatment; (2) tumor resected completely by surgical ablation and neck lymph node dissection (if necessary); and (3) availability of complete follow-up data, including survival, metastasis, and cause of death. Patients who met the following conditions were excluded: (1) patients with incomplete clinical and laboratory data; (2) patients who received chemotherapy or radiotherapy prior to surgery; (3) patients who were diagnosed with nonsquamous carcinoma, such as adenoid cystic carcinoma and mucoepidermoid carcinoma; and (4) patients with blood and lymphatic system disorders.

### Data Collection

Patients underwent standard workups according to the OSCC clinical pathway. Before the operation, a record of a clear medical history, a complete physical examination, results of laboratory and hematological investigations, results of cone beam computed tomography (CBCT) or head and neck computed tomography, and chest radiographs were obtained. Tumors were excised with adequate margins under intraoperative frozen-section control, and pathological TNM classification was performed according to the American Joint Committee on Cancer (AJCC) Staging Manual (7^th^ Edition).

For each patient, the following information was obtained: age, sex, T stage, N stage, M stage, relapse, lesion site, smoking status, and survival status. The lymphocyte count, monocyte count, neutrophil count, and white blood cell count were retrieved from preoperative blood tests; the NLR, LMR, NWR, and LWR were further derived from these values. All patients were regularly followed up bimonthly until August 31, 2018. Recurrence was defined as the presence of tumors with similar histological characteristics after treatment. Metastasis was defined as tumor recurrence within distant organs.

### Ethics

This study was approved by the Ethics Committee of Nanjing Stomatological Hospital, Medical School of Nanjing University(2015NL-018KS), and written informed consent was obtained from the patients or their families.

### Statistical Analysis

The endpoints of this study included OS (time between diagnosis and death from any cause), DFS (time between end of primary treatment to recurrence/second primary/last follow-up), and MFS (time between diagnosis and the occurrence of distant metastasis).

The associations between the NLR, LMR, NWR, LWR, and clinicopathological parameters were evaluated by the Chi-square test. The continuous variables NLR, LMR, NWR, and LWR were analyzed as dichotomous variables according to the optimal cutoff value. The associations of the NLR, LMR, NWR, and LWR with the state of metastasis were judged by Student’s t test. Patients’ clinical endpoints were calculated using Kaplan-Meier curves and compared by the log-rank test. Backward stepwise multivariate Cox proportion analysis was performed to determine the influence of age, sex, TNM stage, nodal status, metastasis, smoking, NLR, LMR, NWR, and LWR on OS, DFS, and MFS. The results from the Cox analysis are reported as relative risks with the corresponding 95% confidence intervals (CIs). Statistical analyses were performed with SPSS software (version 19, SPSS Inc., Chicago, IL, USA). *P* < 0.05 was considered statistically significant.

## Results

### Patient Characteristics

A total of 493 patients fulfilled the inclusion criteria of this study, and all their clinicopathological characteristics are presented in **Table 1**. Briefly, there were a total of 261 males and 232 females; among whom 208 patients were <60 years old, 285 patients were ≥ 60 years old, and 132 patients had a smoking habit, and 361 patients did not. According to tumor stage, a total of 164 (33.3%) cases were T1 (tumor diameter ≤ 2 cm), 246 (49.9%) were T2 (2 cm < tumor diameter ≤ 4 cm), 34 (6.9%) were T3 (tumor diameter > 4 cm), and 49 (9.9%) were T4 (the tumor spread to the surrounding structure). Among the 493 patients, 30 had local relapse, and 44 had distant metastasis. Until the last follow-up, 416 (84.4%) patients remained alive, whereas 77 (15.6%) patients died due to disease recurrence, metastasis, or other reasons. All data are shown in the **Table 1**.

**Table 1:** Clinico-pathological characteristics of patients with OSCC.

Characteristics	*N* = 493	Percentage (%)
Age (years)		
<60	208	42.2
≥60	285	57.8
Gender		
Male	261	52.9
Female	232	47.1
Smoking		
Yes	132	27
No	361	73
Tumor size		
T1	164	33.3
T2	246	49.9
T3	34	6.9
T4	49	9.9
Lymph node status		
N0	352	71.4
N1	88	17.8
N2	53	10.8
N3	0	0
Relapse		
Yes	30	6.1
No	463	93.9
Metastasis		
Yes	44	8.9
No	449	91.1
Site of the lesions		
Tongue	218	44.2
Buccal mucosa	121	24.6
Gingiva	88	17.8
Floor of the mouth	34	6.9
Palate	20	4.1
Lip	12	2.4
Survival status		
Alive	416	84.4
Dead	77	15.6

### Associations of Serum Biomarkers With Clinicopathological Characteristics of Patients With OSCC

In the present study, patients were stratified using optimum cutoff values for the NLR (2.9), LMR (3.4), NWR (0.67), and LWR (0.23), which were determined according to the highest χ^2^ value defined by Kaplan-Meier survival analysis and log-rank tests. The associations of the above hematological parameters with the clinicopathological characteristics of patients with OSCC are shown in **Table 2**. Older age was associated more with a high NLR (*P*=0.01) and low LWR (*P*=0.005). Males were more strongly associated with a high NLR (*P*=0.01), high NWR (*P*=0.031), low LMR (*P*<0.001), and low LWR (*P*=0.003) than females. Both a high NLR and high NWR were demonstrated to be associated with the presence of metastasis (*P*=0.031 and *P*<0.001, respectively), while a low LWR was demonstrated to be associated with the presence of metastasis (*P*=0.012).

**Table 2:** Associations of NLR, LMR, NWR, and LWR with clinicopathological characteristics.

Parameter	NLR			LMR			NWR			LWR		
	Low n (%)	High n (%)	*P*-Value	Low n (%)	High n (%)	*P*-Value	Low n (%)	High n (%)	*P*-Value	Low n (%)	High n (%)	*P*-Value
Age (years)												
< 60	168 (45.5)	40 (32.3)	**0.01**	45 (436.3)	163 (44.2)	0.124	165 (44.7)	43 (34.7)	0.05	39 (31.5)	169 (45.8)	**0.005**
≥ 60	201 (54.5)	84 (67.7)		79 (63.7)	206 (55.8)		204 (55.3)	81 (65.3)		85 (68.5)	200 (54.2)	
Gender												
Male	183 (49.6)	78 (62.9)	**0.01**	87 (70.2)	174 (47.2)	**<0.001**	185 (50.1)	76 (61.3)	**0.031**	80 (64.5)	181 (49.1)	**0.003**
Female	186 (50.4)	46 (37.1)		37 (29.8)	195 (52.8)		184 (49.9)	48 (38.7)		44 (35.5)	188 (50.9)	
Tumor size												
T1-T2	313 (84.8)	97 (78.2)	0.089	102 (82.3)	308 (83.5)	0.093	313 (84.8)	97 (78.2)	0.089	99 (79.8)	311 (84.3)	0.253
T3-T4	56 (15.2)	27 (21.8)		22 (17.7)	61 (16.5)		56 (15.2)	27 (21.8)		25 (20.2)	58 (15.7)	
Lymph node stage												
N0	268 (72.6)	83 (66.9)	0.226	82 (66.1)	269 (72.9)	0.605	264 (71.5)	87 (70.2)	0.769	81 (65.3)	270 (73.2)	0.095
N1-N3	101 (27.4)	41 (33.1)		42 (33.9)	100 (27.1)		105 (28.5)	37 (29.8)		43 (34.7)	99 (26.8)	
Relapse												
No	347 (94.0)	116 (93.5)	0.884	115 (92.7)	348 (94.3)	0.528	347 (94.0)	116 (93.5)	0.884	116 (93.5)	347 (94.0)	0.844
Yes	22 (6.0)	8 (6.5)		9 (7.3)	21 (5.7)		22 (6.0)	8 (6.5)		8 (6.5)	22 (6.0)	
Metastasis												
No	342 (92.7)	107 (86.3)	**0.031**	108 (87.1)	341 (92.4)	0.072	341 (88.1)	108 (75.0)	**<0.001**	106 (85.5)	343 (93.0)	**0.012**
Yes	27 (7.3)	17 (13.7)		16 (12.9)	28 (7.6)		28 (11.9)	16 (25.0)		18 (14.5)	26 (7.0)	

NLR, neutrophil-to-lymphocyte ratio; LMR, lymphocyte-to-monocyte ratio; NWR, neutrophil-to-white blood cell ratio; LWR, lymphocyte-to- white blood cell ratio.

Bold indicates statistical significance.

### Associations of the NLR, LMR, NWR, and LWR With the State of Metastasis and Survival

**Figure 1:**
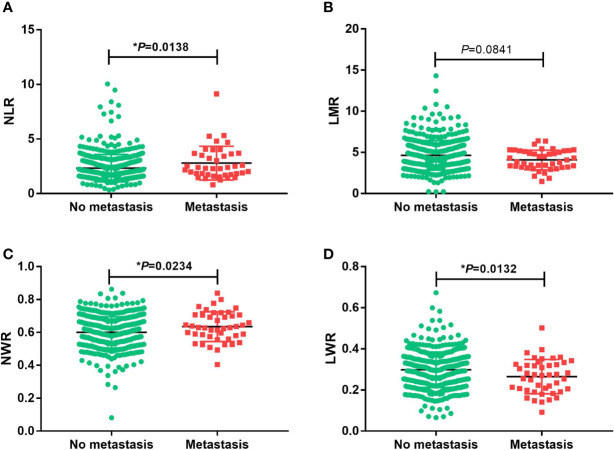
Associations of the prognostic values with the state of metastasis in oral squamous cell carcinoma. **(A)** neutrophil-to-lymphocyte ratio (NLR), **(B)** lymphocyte-to-monocyte ratio (LMR), **(C)** neutrophil-to-white blood cell ratio (NWR) and **(D)** lymphocyte-to-white blood cell ratio (LWR). Asterisks (*) indicate statistical significance.

**Figure 2:**
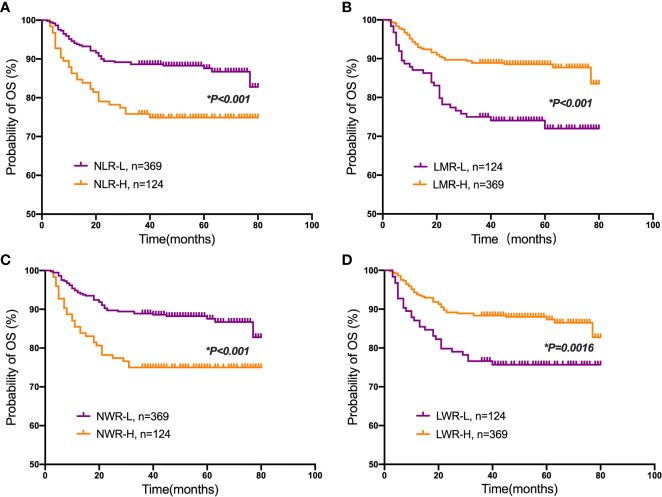
Kaplan-Meier plots for the probability of overall survival (OS). OS rates of the patient subgroups stratified by the **(A)** neutrophil-to-lymphocyte ratio (NLR), **(B)** lymphocyte-to-monocyte ratio (LMR), **(C)** neutrophil-to-white blood cell ratio (NWR) and **(D)** lymphocyte-to-white blood cell ratio (LWR). Asterisks (*) indicate statistical significance.

**Figure 3:**
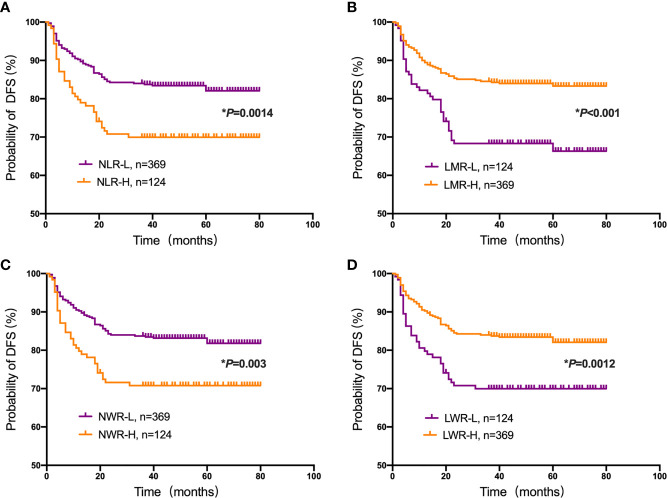
Kaplan-Meier plots for the probability of disease-free survival (DFS). DFS rates of the patient subgroups stratified by the **(A)** neutrophil-to-lymphocyte ratio (NLR), **(B)** lymphocyte-to-monocyte ratio (LMR), **(C)** neutrophil-to-white blood cell ratio (NWR) and **(D)** lymphocyte-to-white blood cell ratio (LWR). Asterisks (*) indicate statistical significance.

**Figure 4:**
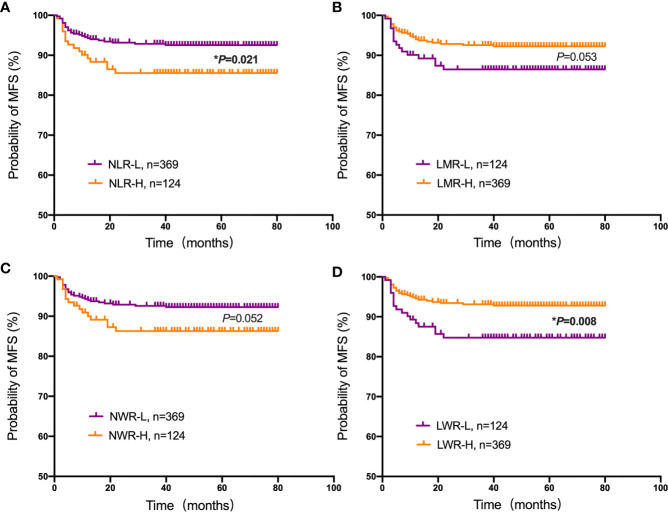
Kaplan-Meier plots for the probability of metastasis-free survival (MFS). MFS rates of the patient subgroups stratified by the **(A)** neutrophil-to-lymphocyte ratio (NLR), **(B)** lymphocyte-to-monocyte ratio (LMR), **(C)** neutrophil-to-white blood cell ratio (NWR) and **(D)** lymphocyte-to-white blood cell ratio (LWR). Asterisks (*) indicate statistical significance.

Student’s t test was used to compare the NLR, LMR, NWR and LWR with the state of metastasis. The results showed that a high NLR and NWR and a low LWR were associated with metastasis (*P*<0.05) (**Figure 1**). To evaluate the prognostic values of the NLR, LMR, NWR, and LWR on OS (**Figure 2**), DFS (**Figure 3**), and MFS (**Figure 4**), the Kaplan-Meier method was used. The analysis of the results revealed that patients with a high NLR (*P*<0.001) or NWR (*P*<0.001) had significantly worse OS, while those with a high LMR (*P*<0.001) or LWR (*P*=0.0016) had better OS. Similar differences were also observed in DFS. **Figure 3** shows that patients with a high NLR (*P*=0.0014) or NWR (*P*=0.003) had significantly worse DFS, while those with a high LMR (*P*<0.001) or LWR (*P*=0.0012) had better DFS. However, with regard to MFS, only the NLR and LWR were significantly associated with MFS. Specifically, patients with a high NLR and low LWR had significantly poor MFS (*P*=0.021 and 0.008, respectively).

### Univariate and Multivariate Analyses of Prognostic Factors

To further verify the prognostic values of the NLR, LMR, NWR, and LWR, univariate and multivariate analyses of OS, DFS and MFS were performed. As shown in **Table 3**, the univariate analysis demonstrated that nodal status (*P*<0.001), metastasis (*P*<0.001), the NLR (*P*=0.001), the LMR (P<0.001), the NWR (*P*=0.001), and the LWR (*P*=0.002) were significantly associated with OS. Subsequently, these parameters were further analyzed with multivariate Cox regression analysis, and the results revealed that nodal status (hazard ratio [HR]=2.912, 95% CI=1.853-4.575, p<0.001), metastasis (HR=8.008, 95% CI=4.988-12.857, *P*<0.001), and the LMR (HR=0.532, 95% CI=0.336-0.840, *P*=0.007) were significantly associated with OS, indicating that nodal status, metastasis, and the LMR are independent prognostic factors for OS.

**Table 3:** Univariate and multivariate analysis for OS.

Parameter	Univariate analysis		Multivariate analysis	
	HR (95%CI)	*P*-Value	HR (95%CI)	*P*-Value
Age (years): ≥60 *vs.* <60	1.505 (0.938-2.414)	0.090		0.215
Gender: female *vs.* male	1.079 (0.690-1.686)	0.740		0.633
Tumor size: T3-T4 *vs.* T1-T2	1.304 (0.752-2.261)	0.345		0.520
Nodal status: N+ *vs.* N0	3.297 (2.106-5.161)	**<0.001**	2.912 (1.853- 4.575)	**<0.001**
Metastasis: Yes *vs.* No	9.371 (5.883-14.925)	**<0.001**	8.008 (4.988-12.857)	**<0.001**
Smoking: Yes *vs*. No	0.834 (0.492-1.414)	0.500		0.205
NLR: ≥2.9 *vs. <*2.9	2.173 (1.378-3.427)	**0.001**		0.437
LMR: <3.4 *vs.* ≥3.4	0.414 (0.263-0.650)	**<0.001**	0.532 (0.336-0.840)	**0.007**
NWR: ≥0.67 *vs. <*0.67	2.196 (1.392-3.463)	**0.001**		0.147
LWR: ≥0.23 *vs.<* 0.23	0.486 (0.308-0.769)	**0.002**		0.677

OS, overall survival; NLR, neutrophil-to-lymphocyte ratio; LMR, lymphocyte-to-monocyte ratio; NWR, neutrophil-to-white blood cell ratio; LWR, lymphocyte-to- white blood cell ratio; HR, hazard ratio; CI, confidence interval.

Bold indicates statistical significance.

Adjusted for age, gender, tumor size, nodal status, metastasis, smoking in logistic regression models.

For DFS, the univariate analysis showed that nodal status (*P*<0.001), metastasis (*P*<0.001), the NLR (*P*=0.002), the LMR (*P*<0.001), the NWR (*P*=0.004), and the LWR (*P*=0.002) were significantly associated with prognosis (**Table 4**). However, only nodal status (HR=1.960, 95% CI=1.316-2.919, *P*=0.001), metastasis (HR=21.561, 95% CI=13.878-33.496, *P*<0.001) and the LMR (HR=0.609, 95% CI=0.406-0.915, *P*=0.017) remained significant, indicating that nodal status, metastasis and the LMR are independent prognostic factors for DFS in OSCC patients.

**Table 4:** Univariate and multivariate analysis for DFS.

Parameter	Univariate analysis		Multivariate analysis	
	HR (95%CI)	*P*-Value	HR (95%CI)	*P*-Value
Age (years): ≥60 *vs.* <60	1.178 (0.788-1.761)	0.424		0.831
Gender: female *vs.* male	1.073 (0.725-1.588)	0.724		0.505
Tumor size: T3-T4 *vs.* T1-T2	1.077 (0.647-1.794)	0.776		0.673
Nodal status: N+ *vs.* N0	2.410 (1.626-3.573)	**<0.001**	1.960 (1.316-2.919)	**0.001**
Metastasis: Yes *vs.* No	24.460 (15.864-37.715)	**<0.001**	21.561 (13.878-33.496)	**<0.001**
Smoking: Yes *vs.* No	0.968 (0.619-1.514)	0.887		0.336
NLR: ≥2.9 *vs. <*2.9	1.914 (1.275-2.872)	**0.002**		0.932
LMR: <3.4 *vs.* ≥3.4	0.460 (0.308-0.686)	**<0.001**	0.609 (0.406-0.915)	**0.017**
NWR: ≥0.67 *vs. <*0.67	1.831 (1.217-2.755)	**0.004**		0.940
LWR: ≥0.23 *vs. <* 0.23	0.520 (0.346-0.780)	**0.002**		0.670

DFS, disease free survival; NLR, neutrophil-to-lymphocyte ratio; LMR, lymphocyte-to-monocyte ratio; NWR, neutrophil-to-white blood cell ratio; LWR, lymphocyte-to- white blood cell ratio; HR, hazard ratio; CI, confidence interval.

Bold indicates statistical significance.

Adjusted for age, gender, tumor size, nodal status, metastasis, smoking in logistic regression models.

The univariate analysis shown in **Table 5** revealed that nodal status (*P*=0.04), the NLR (*P*=0.024) and the LWR (*P*=0.009) were significantly associated with MFS. However, in the multivariate analyses using the Cox proportional hazards model for MFS, only the LWR (HR=0.451, 95% CI=0.247-0.823, *P*=0.009) was identified as a significant prognostic factor.

**Table 5:** Univariate and multivariate analysis for MFS.

Parameter	Univariate analysis		Multivariate analysis	
	HR (95%CI)	*P*-Value	HR (95%CI)	*P*-Value
Age (years): ≥60 *vs.* <60	1.804 (0.944-3.447)	0.074		0.131
Gender: female *vs.* male	1.125 (0.623-2.032)	0.695		0.455
Tumor size: T3-T4 *vs.* T1-T2	0.782 (0.331-1.850)	0.576		0.501
Nodal status: N+ *vs.* N0	1.877 (1.029-3.425)	**0.040**		0.058
Smoking: Yes *vs*. No	0.902 (0.456-1.784)	0.766		0.723
NLR: ≥2.9 *vs. <*2.9	2.008 (1.094-3.685)	**0.024**		0.429
LMR:<3.4 *vs.* ≥3.4	0.551 (0.298-1.019)	0.058		0.641
NWR: ≥0.67 *vs. <*0.67	1.817 (0.983-3.359)	0.057		0.708
LWR: ≥0.23 *vs.<* 0.23	0.451 (0.247-0.823)	**0.009**	0.451 (0.247-0.823)	**0.009**

MFS, metastasis free survival; NLR, neutrophil-to-lymphocyte ratio; LMR, lymphocyte-to-monocyte ratio; NWR, neutrophil-to-white blood cell ratio; LWR, lymphocyte-to- white blood cell ratio; HR, hazard ratio; CI, confidence interval.

Bold indicates statistical significance.

Adjusted for age, gender, tumor size, nodal status, metastasis, smoking in logistic regression models.

## Discussion

OSCC is a highly malignant tumor type (18), and the development of effective methods to diagnose and treat OSCC represents an urgent task (19). Inflammation is a basic and important pathologic response of the body triggered by damage that can occur alone or accompany tumors (20). Carcinogenesis not only recruits WBCs to and around neoplasms but also causes tissue damage through physical and chemical mechanisms, leading to wide, nonspecific inflammatory responses and systemic inflammatory responses (21). Thus, changes the counts and component proportions of white blood cells (WBCs) are changed. In the present study, the clinicopathological characteristics, follow-up data, and various peripheral blood component ratios of 493 patients with OSCC were analyzed to verify the correlations between cancer-associated systemic inflammation and OSCC outcomes. We found that a high NLR and low LWR were associated with older age. We also found that males were more strongly associated with a high NLR, high NWR, low LMR, and low LWR than females, consistent with previous studies (22). In addition, a high NLR, high NWR and low LWR were found to be associated with the presence of metastasis. However, relationships between serum biomarkers and other clinicopathological characteristics, including tumor stage, lymph node stage and relapse, were not found. This suggests that the predictive effect of the NLR, NWR, LMR and LWR may be independent of the TNM staging system. We can use these serum biomarkers to complement the diagnosis of OSCC, to evaluate prognosis and to assess treatment.

Regarding cancer-associated inflammatory responses, a previous study demonstrated a moderate correlation between monocytes and neutrophils, as they both have a negative impact on the prognosis of patients with oropharyngeal cancer, whereas lymphocytes have the opposite effect (23). The NLR, which reflects the balance between a protumor inflammatory status and an antitumor immune status, is the most widely used parameter for prognostic prediction. Most studies have reported that an increased NLR is related to worse disease control and poor survival (24–26). Similarly, in the current study, we also observed that patients with a high NLR were associated with a significant downward trend of survival probability according to the 80-month Kaplan-Meier curves for OS, DFS and MFS. Unfortunately, the multivariate analysis demonstrated that the NLR is not an independent prognostic factor for OS, DFS or MFS. The LWR is another predictive biomarker related to the prognosis of various cancers, including gastric cancer and non-small-cell lung cancer (27, 28). However, we did not find any literature reporting an association between the LWR and OSCC. According to the results of the univariate and multivariate analyses in our study, the LWR is an independent prognostic factor for MFS. This might be the first report to state a cutoff value for the LWR in predicting metastasis and demonstrated that a low LWR is a poor prognostic factor in patients with OSCC. The prognostic value of the LMR has been investigated by many schoolers while studying various cancers, including breast, lung, esophageal, gastric, colorectal, pancreatic, bladder, and cervical cancers (29). Lin et al. studied 256 patients with newly diagnosed metastatic nasopharyngeal carcinoma who received chemotherapy and found that a high LMR was associated with a good prognosis (30). The data of our study demonstrate that the LMR is the only serum biomarker independently related to both OS and DFS (according to the multivariate analysis).

Based on upon evidence, we speculate that the NLR, NWR, and LWR might affect the survival rate in an indirect way (*i.e.*, by relating to poor clinicopathological manifestations or promoting the migration of cancer cells), while the LMR directly reflects patient survival duration after treatment. Although the underlying mechanisms of the relationship between the LMR and prognosis are not well understood, the LMR is thought to reflect the balance between the prognosis-improving effect of lymphocytes and the adverse effect of monocytes.

According to previous studies, the prognosis of OSCC is highly heterogeneous, with an overall 5-year survival rate of approximately 64%, and the median survival duration for patients with locoregionally recurrent or metastatic OSCC is 8~10 months (31, 32). Oral malignancies progress through four stages; in the early stage (stages I and II), the 5-year survival rate is approximately 80%. However, it is reduced to approximately 50% in patients with locoregional metastasis (stages III, IVA, and IVB) and approximately 25% if distant metastasis is present (stage IVC) (33, 34). Therefore, it is of great significance to understand the relationship between metastasis and inflammatory cells in predicting the prognosis of OSCC. In general, metastasis comprises the sequential occurrence of uncontrolled cancer cell proliferation, invasion into the blood or lymph circulation, and crosstalk with various components of the new microenvironment, including parenchymal, stromal and inflammatory cells. However, the precise mechanism of the process has not yet been clarified, and the factors affecting its occurrence are mostly uncertain. Based on the above results, lymph node metastasis is closely correlated with poor survival, and it was also proven in our study that metastasis is an independent predictor for poor survival. In addition, we found that a high NLR and NWR and a low LWR were associated with metastasis, whereas the LWR was an independent prognostic factor for predicting MFS. If these results can be verified by further evidence, patients who have a high risk of metastasis will receive direct benefits.

We enrolled 493 patients in the present study, with the longest follow-up exceeding 80 months. The main strength of the current study was that a large number of patients treated at a single institution were included, with a relatively long follow-up duration, and data were collected by using uniform database templates to ensure consistency, which improved the quality of the evidence. However, some inherent limitations were inevitable because of its retrospective nature. For example, patients who had blood or lymphatic system disorders were excluded because of a strict eligibility criterion, which may have caused patient selection bias. In addition, the therapy strategies were not uniform but varied based on the patient’s condition, and the effect of different treatment-related factors on prognosis was not evaluated. Therefore, a prospective study designed to confirm the prognostic value of the pretreatment NLR, LMR, NWR, and LWR is needed. Despite the limitations of this study, pretreatment serum biomarkers can be quick, simple, easily obtainable, and cost-effective tools to predict the outcome of OSCC.

## Conclusion

The results of this study showed that OSCC patients with a high LMR and low NWR had prolonged OS and DFS, while a high NLR and low LWR were associated with poor OS, DFS and MFS. Moreover, once the prognostic significance of these novel markers is defined and verified by researchers, they can be widely applied in the clinic and help doctors identify patients at high risk for disease recurrence and tumor progression.

## Data Availability Statement

The original contributions presented in the study are included in the article/supplementary material. Further inquiries can be directed to the corresponding authors.

## Ethics Statement

The studies involving human participants were reviewed and approved by Ethics Committee of Nanjing Stomatological Hospital, Medical School of Nanjing University. The patients/participants provided their written informed consent to participate in this study.

## Author Contributions

The authors contributed in the following manner: Study conception and design: YM and YN. Acquisition of data: YS, JJ, and MT. Statistical analysis: MD, YS, and CZ. Manuscript preparation: MD. English editing: QL. Critical manuscript revision: LD and HD. MD and YS contributed equally to this work. All authors contributed to the article and approved the submitted version.

## Funding

The authors are grateful for grants from the National Natural Science Foundation of China (Nos. 81371680, 81571800, and 81772880), the Development of Science and Technology of Nanjing (No. 201803036), Jiangsu Provincial Medical Talent (No. ZDRCC2016016), the Nanjing Medical Science and Technique Development Foundation (QRX17083, ZKX18035), YKK18124, YKK20151, and YKK19094).

## Conflict of Interest

The authors declare that the research was conducted in the absence of any commercial or financial relationships that could be construed as a potential conflict of interest.

## Publisher’s Note

All claims expressed in this article are solely those of the authors and do not necessarily represent those of their affiliated organizations, or those of the publisher, the editors and the reviewers. Any product that may be evaluated in this article, or claim that may be made by its manufacturer, is not guaranteed or endorsed by the publisher.

## Abbreviations

OSCC, oral squamous cell carcinoma; NLR, neutrophil-to-lymphocyte ratio; LMR, lymphocyte-to-monocyte ratio; NWR, neutrophil-to-white blood cell ratio; LWR, lymphocyte-to-white blood cell ratio; OS, overall survival; DFS, disease-free survival; MFS, metastasis-free survival; T, tumor; N, nodal metastasis; M, distant metastasis; CBCT, cone beam computed tomography; AJCC, American Joint Committee on Cancer; WBCs, white blood cells; HR, hazard ratio; CI, confidence interval.
